# The Role of Carotenoids in Human Skin

**DOI:** 10.3390/molecules161210491

**Published:** 2011-12-16

**Authors:** Maxim E. Darvin, Wolfram Sterry, Juergen Lademann, Theognosia Vergou

**Affiliations:** 1 Department of Dermatology, Venerology and Allergology, Center of Applied and Cutaneous Physiology (CCP), Charité University Medicine Berlin, Charitéplatz 1, Berlin 10117, Germany; 2 A. Sygros’ Hospital, Department of Dermatology, University of Athens, Athens 16121, Greece

**Keywords:** β-carotene, lycopene, antioxidants, free radicals, ageing, fruit, vegetables

## Abstract

The human skin, as the boundary organ between the human body and the environment, is under the constant influence of free radicals (FR), both from the outside in and from the inside out. Carotenoids are known to be powerful antioxidant substances playing an essential role in the reactions of neutralization of FR (mainly reactive oxygen species ROS). Carotenoid molecules present in the tissue are capable of neutralizing several attacks of FR, especially ROS, and are then destroyed. Human skin contains carotenoids, such as α-, γ-, β-carotene, lutein, zeaxanthin, lycopene and their isomers, which serve the living cells as a protection against oxidation. Recent studies have reported the possibility to investigate carotenoids in human skin quickly and non-invasively by spectroscopic means. Results obtained from *in-vivo* studies on human skin have shown that carotenoids are vital components of the antioxidative protective system of the human skin and could serve as marker substances for the overall antioxidative status. Reflecting the nutritional and stress situation of volunteers, carotenoids must be administered by means of antioxidant-rich products, e.g., in the form of fruit and vegetables. Carotenoids are degraded by stress factors of any type, *inter alia*, sun radiation, contact with environmental hazards, illness, *etc.* The kinetics of the accumulation and degradation of carotenoids in the skin have been investigated.

## 1. Introduction

Free radicals (FR) are highly reactive molecules formed as a result of metabolic processes [1,2,3,4]. They play an important signaling function in the human organism [1,5] as well as acting against viruses and bacteria [6,7]. In addition, there is an ample variety of external factors leading to the formation of FR and especially reactive oxygen species (ROS) in human skin. The solar UV radiation is one of these factors [8,9,10], but pollutants can also induce ROS formation upon contact with human skin [11,12]. If the concentration of FR in tissue exceeds a critical value both cell and cell components are destroyed, entailing tissue damage and even cancer [13,14,15,16,17].

Human skin is continuously exposed to FR. The latest findings of Zastrow *et al.* have shown that it is not only the solar UV radiation, which induces the formation of FR (mainly ROS) in human skin [18], but also the visible and infrared range of the spectra [19]. Utilizing the antioxidative protective system, the human body has developed a complex defence mechanism against the harmful effects of these highly reactive substances [20,21,22]. The antioxidants in human skin include, *inter alia*, the vitamins, the carotenoids, and a variety of enzymes [23,24,25,26,27,28]. Most of these substances cannot be produced by the human body independently, but must be taken up with food rich in carotenoids, for instance, with fruit and vegetables [29]. The digestion and metabolism of dietary carotenoids by humans is a complex process [30], which could be genetically dependent [31]. After digestion, the carotenoids accumulate in high amounts in the adipose tissue, liver and the blood [32].

There are two main pathways for the accumulation of carotenoids in the epidermis—Diffusion from the adipose tissue, blood and lymph flows; and the secretion via sweat glands and/or sebaceous glands onto the skin surface and their subsequent penetration [33,34]. The group of carotenoids available in human skin includes α-, γ-, β-carotene, lycopene, lutein, zeaxanthin and their isomers [35]. They are known as potent quenchers of singlet oxygen [36] and other FR [37] in biological systems. Recent investigations have shown that the carotenoids could serve as marker substances for the entire antioxidative network of human skin [38,39]. This is due to the fact that the antioxidants form protective chains in the human tissue, acting synergistically, in order to protect each other against the destructive action of the FR and in particular ROS [38,40,41,42]. If any of the substances in this chain are detected, information about the other components of the antioxidative protective system is automatically provided. However, it has to be taken into account that the kinetics of the individual components of the antioxidants could differ during accumulation and degradation.

So far, carotenoids in biological samples have mainly been analyzed by High Pressure Liquid Chromography (HPLC) [43]. This process requires biopsies taken from tissue and blood. The samples taken are then prepared and analyzed. As this invasive measuring method requires a high number of biopsies, and it is unsuitable for analyzing the kinetics of the carotenoids in human skin.

In recent years, it has become possible to detect carotenoids in human skin non-invasively, selectively and highly sensitively using optical methods [44,45,46,47]. In the following paragraphs the non-invasive techniques used in the studies for measurement of carotenoids in human skin are summarized.

Carotenoids are Raman active molecules characterized by three prominent Stokes lines at 1005 cm^−1^ (rocking motion of the methyl group), 1156 cm^−1^ (carbon-carbon single-bond stretch vibration of the conjugated backbone) and 1523 cm^−1^ (carbon–carbon double-bond stretch vibration of the conjugated backbone). Namely, the C=C bonds are responsible for the action of carotenoids as antioxidants [48,49]. Based on the absorption properties of carotenoids, the resonance excitation should be performed in the blue-green range of the optical spectrum. The intensity of the prominent Raman line at 1523 cm^−1^, which originated under excitation at 488 nm and 514.5 nm, was measured for determination of carotenoid concentrations in human skin [50,51]. The high fluorescence background of human skin was substantially reduced by the use of a photo bleaching effect [52,53]. The resonance Raman spectroscopy (RRS) based system utilized by our group has been described in detail previously [45].

Another possibility is Raman microscopy (RM). The *in vivo* Raman microscopic measurements were performed using the skin composition analyzer (River Diagnostics, Model 3510, Rotterdam, The Netherlands) in the fingerprint range between 400 and 1800 cm^−1^. The utilized excitation wavelength was 785 nm, which permitted the investigation of the deep-located skin areas. The carotenoids were measured non-resonantly by the intensity of the corresponding Stokes line at 1523 cm^−1^, from the skin surface down to a depth of 30 µm in 2 µm increments. This method has previously been described in detail by Caspers *et al.* [54,55].

Dermal carotenoids can also be measured with reflection spectroscopy (RS) [47,56]. For this purpose, the LED-based miniaturized spectroscopic system (MSS) was developed. A LED emitted bright spectrum in the range between 440 and 490 nm was sufficient to overlap the absorption of carotenoids. The backscattered signal provides information about the carotenoid concentration. The small dip in the diffusely reflected spectrum measured in the range between 458 and 472 nm, is correlated to the concentration of carotenoids in human skin. The RS measurements could only be performed on the palm or heel areas, where the epidermis is thick enough and the influence of other skin chromophores is negligible. A comparison between the results of the MSS with those of the RRS yielded an excellent correlation. Contrary to the relatively large Raman system, the size of the MSS is that of a computer mouse and can be directly controlled by the Bluetooth system of a laptop or mobile phone. The utilized MSS for measurement of carotenoids in human skin has been previously described in detail by our group [57].

*In-vitro* measurements of carotenoids can also be performed with these optical methods. For example, porcine ear skin does not contain a high amount of carotenoids, as only a very low concentration lies near the detection limit. Otherwise, bovine udder skin contains a high concentration of carotenoids and is well suited as an *in-vitro* model for measurement of carotenoids in the skin [58].

In the present review paper the *in-vivo* investigations carried out at the Center of Experimental and Applied Cutaneous Physiology (CCP) at the Department of Dermatology of the Charité - Universitätsmedizin Berlin are described. In various studies different volunteers were investigated. The investigations were carried out on the palm, forehead, forearm and back of healthy volunteers aged between 20 and 70 years with skin types II or III in accordance with the Fitzpatrick classification [59]. All volunteers had normal skin without visible abnormalities, such as extremely dry or fatty skin, wounds, or skin diseases. All studies had been approved by the Ethics Committee of the Charité - Universitätsmedizin, Berlin. The importance of the related results to medicine, cosmetology and dietary sciences is discussed.

## 2. Results and Discussion

### 2.1. *In Vivo* Analysis of the Concentration of Carotenoids in Human Skin

**Figure 1:**
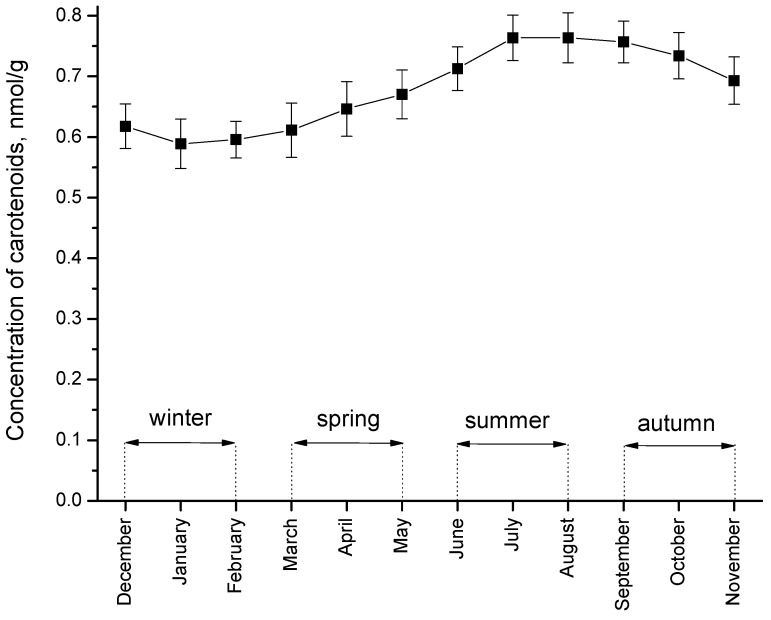
Average monthly values of the dermal carotenoid concentration for one volunteer during a one-year period measured on the inner palm.

After the development of the RRS for the *in vivo* detection of carotenoids in human skin, the system was tested on volunteers at the CCP during a one-year study. Every day before having lunch, the volunteers placed their hands on the sensor head and the carotenoid concentration was measured in the palm region. The entire measurement took only a few seconds. The measurements were complemented by questionnaires, in which the volunteers gave information about their daily diet and stress factors. As a result of these measurements it could be clearly demonstrated that the concentration of the dermal carotenoids represented a fingerprint of the dietary habits and stress situations of the volunteers. Smokers and volunteers with unhealthy food habits exhibited very low values, whilst the volunteers with a healthy diet and moderate stress showed high carotenoid values. Moreover, the results for all volunteers demonstrated that the antioxidant concentration was higher by approximately 25% in summer and autumn than in winter and springtime (“seasonal increase”, p = 0.001). This may be due to the fact that higher amounts of fruit and vegetables are consumed in the summer and autumn months [60]. Also, there are indications that the degree of freshness and ripeness of the products could play a decisive role [61,62]. Whereas in summer and autumn the fruit and vegetables consumed by the volunteers were supplied by regional producers, these products were imported from Latin America or Asia in winter and springtime. Figure 1 shows a “seasonal increase” in the concentration of carotenoids in the skin of one volunteer, obtained during a one-year period, which is typical among all volunteers participating in the study.

Under constant dietary and stress conditions, the carotenoid concentration in the skin remains constant for a considerable length of time. Diseases, for instance, such as the common cold, lead to a strong degradation of carotenoids in human skin [60]. Smoking also reduces the concentration of carotenoids in human skin [45,63]. On the other hand, a change in the nutritional behaviour, for instance, an increased intake of fruit and vegetables as well as carotenoid-rich supplements, result in an increased concentration of cutaneous carotenoids [64,65,66,67], which may well protect the skin against oxidative stress [36,68]. In the present study it could be shown that the nutritional behaviour and stress situation of the volunteers are well reflected by the concentration of dermal carotenoids. These results are in agreement with the results obtained by other groups [69,70,71].

### 2.2. Distribution of Carotenoids in Human Skin

**Figure 2:**
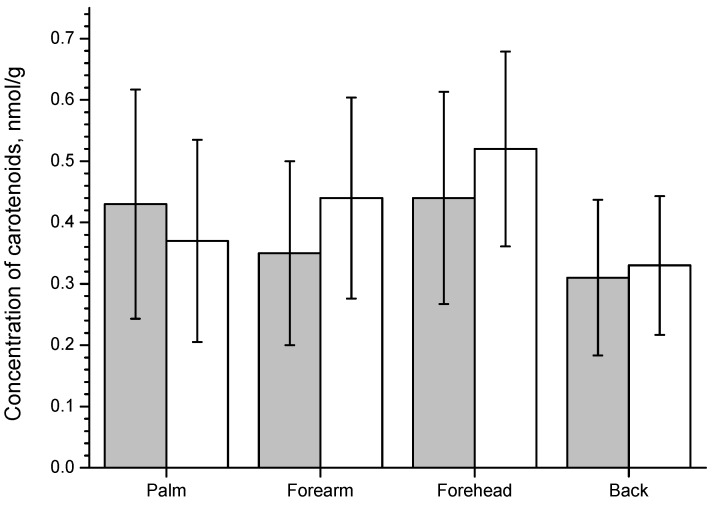
Concentration of β-carotene (grey column) and lycopene (white column) on different body sites measured with resonance Raman spectroscopy.

The distribution of cutaneous carotenoids in human skin, measured by RRS, depends strongly on the skin area examined (forehead, palm, forearm, back) and drastically changes inter-individually [72]. Figure 2 shows the average value ± standard deviation for dermal beta-carotene and lycopene measured on different body sites.

The spatial distribution of carotenoids in the epidermis within one skin area was investigated by the use of RM. It was found that carotenoids distributed non-homogeneously showing a prominent maximum close to the skin surface, lying in a depth of approx. 4–8 µm [46]. Subsequently, the concentration of carotenoids continuously decreased, at least until the measured depth of 30 µm, appropriated for RM measurements [33]. Topical application of cosmetic formulations containing carotenoids, give rise to their increase in the stratum corneum [46,73], which could be investigated with both the RRS and RM methods.

The highest concentration of carotenoids measured in the top layer of epidermis, in stratum corneum, could be explained by the delivery of fat-soluble carotenoids with sebum and/or sweat secretion on the skin surface. Reaching the skin surface, carotenoids penetrate inside the epidermis like topically applied substances, thus increasing their concentration in superficial areas [33,46]. The same mechanism was previously observed by Thiele *et al.* for vitamin E [74].

### 2.3. Factors Influencing the Concentration of Carotenoids in Human Skin

In various follow-up studies the influence of different stress factors on the dermal antioxidative status was investigated. It is known from the literature that the solar UV radiation could lead to the formation of FR in human skin [8,75], which at high concentrations are capable of damaging the antioxidative network [76]. Therefore, the volunteers were exposed to a minimal erythema dose (MED) emitted by a sun simulator. The MED is the radiation dose necessary to induce mild sunburn in the irradiated volunteers. The carotenoid measurements taken prior to and after UV irradiation for a period of four days showed that after irradiation, the β-carotene concentration remained constant for a period of 30 to 60 min, but subsequently declined drastically. Contrary to the β-carotene, the lycopene concentration dropped strongly immediately after irradiation [76]. This is comprehensible as lycopene is a highly efficient antioxidant, whose quenching rate constant in the reaction of neutralization of FR (mainly ROS) is higher in comparison to other carotenoids [77,78]. The original level of the carotenoids was restored not before 2 to 4 days, depending on the individual volunteer. Application of tissue tolerable plasma on human skin, which is used for microbial disinfection, reduces the concentration of carotenoids in the epidermis because of the formation of FR (mainly ROS), subsequent to UVB radiation (310 nm). The carotenoid concentration was markedly reduced in the upper part of the stratum corneum at least to a depth of 10 µm, which was measured with RM [79].

In another study, infrared radiation as used in clinical and private applications was investigated. Here also, a strong degradation of both β-carotene and lycopene was observed [80,81,82]. Whereas, it is known that the UV radiation could induce FR and especially ROS formation, which at increased doses may even destroy the antioxidants in the skin, this effect is surprising in the case of the infrared spectral range, due to the fact that the energy of the photons in this range is insufficient to form FR directly.

By means of electron spin resonance it was possible to demonstrate that, indeed, human skin is subjected to a radical formation process subsequent to infrared irradiation, as a consequence of which the antioxidants are destroyed [83,84]. Heat shock-induced radicals and/or enzymatic processes are probably involved in the transfer of the energy of IR quanta in the skin [85]. Thus, human skin must have structures, which absorb and accumulate the energy of the photons and then induce radical formation. Mitochondria, e.g., are known for such processes. The results of this study are in good agreement with the findings of Zastrow *et al.*, who determined the FR action spectrum for the whole spectrum of the solar radiation [19]. The results showed that 50% of the FR in the skin are generated by solar UV radiation, whereas the remaining 50% are produced in the visible and infrared spectral ranges.

We strongly believe that these results will influence the development of sunscreens. It is assumed that future sunscreens will not only provide UV protection, but also photo protection in general, which could partially be based on the utilization of antioxidants [73,81,86,87].

### 2.4. Carotenoids and Skin Aging

Resulting from a variety of studies performed at the CCP with the RRS it was established that individuals with high carotenoid concentrations in their skin looked young for their age, whilst individuals with low carotenoid concentrations appeared older. This very subjective observation was objectively investigated in another study, for which purpose the skin was measured using optical skin surface topography [88]. The dermal roughness determined with this system is characterised by the density and depth of furrows and wrinkles, thus serving as an objective criterion for skin aging. This parameter is measured non-invasively, whereby the measurements were taken on the light-exposed skin area of the forehead. The same area was measured also for its carotenoid concentration using the RRS. Originally, within the study, it had been intended to investigate volunteers of the same age. For this purpose, volunteers at 40 years of age were to be recruited, who already exhibited considerable skin aging and who had not changed their lifestyle for years. Consequently, smokers, who had meanwhile desisted from smoking, e.g., were excluded from the study. However, it proved to be very difficult to find a large number of volunteers who had constantly kept their lifestyle unchanged. Therefore, the age segment was extended, now ranging from 40 to 50 years. Following the successful completion of this study, it was analyzed whether the dermal roughness, which is a measure of skin aging, correlated with the age of the volunteers. If the group of volunteers had included persons at different ages, *i.e.*, between 18 and 80 years, it would have been expected, of course, that age indeed influenced the skin surface structure. In the present study it could be shown, however, that no correlation exists between age and skin aging. This is not surprising because the age segment investigated was very narrow, compared to the actual age of the volunteers. However, after comparison of the dermal roughness with the concentration of carotenoid lycopene in the skin, a clear correlation was found showing that individuals with a high lycopene concentration in their skin exhibited less dermal roughness [89]. These objective findings proved the accuracy of the earlier subjective observation. Consequently, a healthy diet, rich in antioxidants including carotenoids, could serve as the best preventive strategy against skin aging. However, it was found that it is impossible to recover one’s youthful appearance by eating increased amounts of fruit and vegetables. Once induced, skin damage cannot be repaired subsequently by changing one’s lifestyle. In any case, a healthy diet is also advisable for older people as it has a positive effect on the years to come.

### 2.5. Topical and Systemic Application of Antioxidants

**Figure 3:**
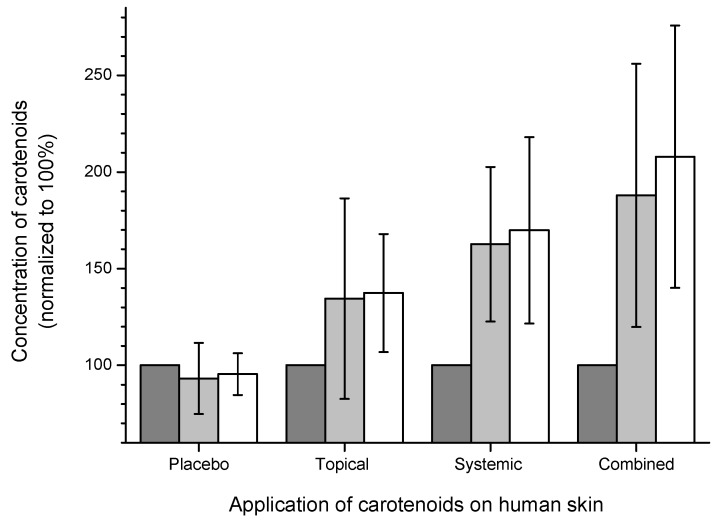
Comparison of the carotenoid concentration on the cheek before treatment (black column), after 4 weeks (grey column) and after 8 weeks (white column) of treatment with carotenoid-containing supplements.

In the previous studies, it could be demonstrated that high antioxidant concentrations in human skin present the best protection strategy against skin aging [24,39]. In follow-up studies methods for the accumulation of the carotenoids in human skin were investigated. In general, there are two possibilities of inducing carotenoid accumulation, one being systemic administration by carotenoid-rich food or intake of food supplements, and the other being topical application of antioxidants in the form of creams and lotions. In a comprehensive study involving several groups of volunteers, the effects of placebo and carotenoid-containing verum tablets were compared to a placebo cream and a verum cream. These products were applied both individually and in a combined form. In the investigations, no increase in the carotenoid concentration in human skin was detected when the placebo products were applied. On the other hand, verum products administered both as cream and as tablets, resulted in an up to 100% increase in the dermal carotenoid concentration of individual volunteers. The maximum accumulation of the carotenoids in human skin was achieved by combined administration of the verum tablets and the verum cream, which however, fell below the sum of the carotenoid concentrations of the two individual applications [86]. This is obviously due to the fact that the systemically administered carotenoids escape onto the skin surface with the sweat and the sebum, spriting there and penetrating into the skin like topically applied [90]. In this process, the reservoir of the horny layer, the stratum corneum, plays an important role. Should this reservoir have already been saturated by a topically applied cream, the sweat cannot penetrate optimally into the stratum corneum, thus rendering the accumulation of the systemically applied carotenoids less efficient [86]. Obtained results are summarized on Figure 3.

The results of this study clearly show, that both the systemic and the topical application of antioxidants lead to an accumulation of these substances in the human skin. For topical application, which can produce the best results, it is essential that the topically applied component is well adapted to the one systemically administered.

### 2.6. Pro-Oxidative Action of Carotenoids and Its Prevention

It should be taken into consideration that carotenoids can also possess a pro-oxidative action in human tissue [91,92]. This action depends on the carotenoid concentration, oxygen tension and the surrounding substances.

The pro-oxidative action of carotenoids is usually observed at relatively high concentrations of applied carotenoids [26,93,94], which strongly exceed the doses of normal dietary intake, the concentrations presented in fruit and vegetables and the physiological concentration in the cells [91]. Moreover, the oxygen tension in the tissue could highly influence the anti- and pro-oxidant properties of carotenoids. The high oxygen pressure gives rise to the pro-oxidative action of carotenoids in biological tissue [95,96]. Under normal physiological conditions, high oxygen tensions could be reached in the lung, but not in the skin [91,97]. The other aspect is the presence of other antioxidants (for example vitamins C and/or E), which possess a synergistic action and could substantially reduce the pro-oxidative action of carotenoids under the above-mentioned conditions [42,91,98,99].

In healthy individuals, a balanced nutrition including antioxidant-rich food, such as fruit and vegetables, could warrant the absence of a pro-oxidative action of carotenoids on the tissue and especially on human skin. Using this approach, any chance of reaching the critical concentration of single antioxidants is excluded [100]. The application of carotenoid-containing supplements or extracts containing balanced physiological concentrations and compositions of antioxidants could serve as an alternative to fruit and vegetables.

### 2.7. Analysis of the Antioxidative Status in Clinical Practice

The aforementioned studies address in particular nutritional sciences and cosmetology. However, the analysis of the carotenoids in human skin is also directly applied in the clinical sector; the analysis of side effects during chemotherapy being just one example [101]. Currently, a wide range of highly efficient chemotherapeutics exists, many of which entail side effects [102,103,104]. Such side effects are often due to the fact that part of the chemotherapeutics or metabolites penetrate together with the sweat out onto the skin surface, where they sprite and penetrate into the skin, again like topically applied substances [105]. Since the reservoir of the stratum corneum is very large and the density of the sweat glands is high on the palms of the hand and the heels of the foot, the penetrated substances are accumulated there to an increased extent. This leads to the development of a hand-foot-syndrome, an inflammatory symptom, the severest degree of which is open wounds [105]. The effect of the chemotherapeutics on these skin areas is often due to radical forming processes. The systemic administration of antioxidants is contra-productive, as it would reduce the effect of the chemotherapeutics in the tumour. Therefore, a prevention strategy was developed based on the fact that the skin is provided with antioxidants through a specific preventive cream, containing a balanced mixture of antioxidants including carotenoids. This cream was applied prior to and during the chemotherapy. Consequently, it is very important to analyze the antioxidative status of the skin prior to and during chemotherapy. This is an essential field of application for RRS.

## 3. Conclusions

Results obtained *in-vivo* and non-invasively show that the concentration of carotenoids in human skin reflects the current lifestyle conditions of volunteers. High concentrations of carotenoids in the skin of volunteers are usually associated with a lifestyle free of stress and a carotenoid-rich supplementation. Low carotenoid concentration is usually attributed to an unhealthy lifestyle, as well as nutrition, illness and smoking. The kinetics of the degradation of dermal carotenoids, subsequently influencing stress factors (sun radiation, illness, fatigue, *etc.*) is a relatively fast process, lasting up to a number of hours, in order to reach maximal degradation, whilst the subsequent recovery is a more prolonged effect, which requires a number of days before levelling.

The importance of the obtained results, with the focus on dermal carotenoids, medicine, cosmetology, dietary sciences and development of protection strategies based on topical, systemic and the combined application of antioxidants are discussed.

The results described and discussed in this review show that optical technologies are excellently suited to determine the antioxidative status in human skin non-invasively, selectively and sensitively, using carotenoids as marker substances. As research and development in the field of optical and spectroscopic systems, as well as miniaturization of light sources and spectrometers is progressing, more efficient, smaller and simpler measuring systems will soon be available on the market at reasonable prices.
